# Clinical Application and Feasibility of MRI-Guided Breast Biopsy of Breast Minimal Lesions in Chinese Population

**DOI:** 10.3389/fonc.2020.00257

**Published:** 2020-03-06

**Authors:** Jie Wang, Ying Song, Jiaqi Liu, Xiangzhi Meng, Zeyu Xing, Menglu Zhang, Feng Ye, Xin Wang, Xiang Wang

**Affiliations:** ^1^Department of Ultrasound, National Cancer Center/National Clinical Research Center for Cancer/Cancer Hospital, Chinese Academy of Medical Sciences and Peking Union Medical College, Beijing, China; ^2^Department of Diagnostic Radiology, National Cancer Center/National Clinical Research Center for Cancer/Cancer Hospital, Chinese Academy of Medical Sciences and Peking Union Medical College, Beijing, China; ^3^Department of Breast Surgical Oncology, National Cancer Center/National Clinical Research Center for Cancer/Cancer Hospital, Chinese Academy of Medical Sciences and Peking Union Medical College, Beijing, China

**Keywords:** breast cancer, minimal lesions, MRI-guided, biopsy, localization

## Abstract

**Objectives:** Some breast lesions are not visible on mammography or ultrasonography, and magnetic resonance imaging (MRI) become the only way to monitor these lesions. The purpose of this study was to evaluate the clinical application of MRI-guided biopsy and MRI-guided wire localization of breast minimal lesions in Chinese population.

**Methods:** We evaluated 95 patients (the most patients of known in China) from August 2013 to December 2017. All the patients were scanned with a 1.5-Tesla MRI system (GE Medical Systems, America) in the prone position using a bilateral 8-channel phased-array breast coil and underwent MRI-guided wire localization or MRI-guided biopsy.

**Results:** MRI-guided wire localization and MRI-guided biopsy were successfully performed in 87 patients with 88 lesions (100%, 88/88). After biopsy or surgery, 36 of 88 lesions (40.91%) were malignant, and 52 of 88 lesions (59.09%) were benign. Thirty-nine of 88 lesions (44.32%) were masses, and 49 of 88 (55.68%) showed non-mass enhancement. Statistical analysis showed there was no significant correlation between the malignancy rate and the type of lesion on MRI (*P* = 0.27). In this study, the rate of malignancy for Breast Imaging-Reporting and Data System (BI-RADS) 5 lesions was 100% (2 of 2) compared with 44.44% for BI-RADS 4C lesions (4 of 9), 42.42% for BI-RADS 4B lesions (14 of 33), and 36.36% for BI-RADS 4A lesions (16 of 44).

**Conclusions:** MRI-guided wire localization with subsequent surgical biopsy and MRI-guided biopsy are safe and effective tools for breast minimal lesions.

## Introduction

Breast cancer is one of the most common cancer diagnosed among women worldwide, accounting for the second leading cause of cancer-related death among women in US and the sixth in Chinese women (1–3). Magnetic resonance imaging (MRI) is widely used as a diagnostic tool for breast imaging in daily practice due to its high sensitivity for detecting primary, recurrent, and residual breast cancer. Some breast lesions are not visible on mammography or ultrasonography, and MRI can be used to regularly monitor these lesions. For suspicious breast lesions that are visible only on MRI, MRI-guided breast biopsy and MRI-guided wire localization with subsequent surgical biopsy are necessary for diagnosis and excluding multi-foci lesions in breast conserving surgery. Although MRI has a high reported sensitivity for detecting breast cancer of 94–100%, its specificity is relatively low, ranging from 37 to 72% (4, 5); thus, biopsy is often required to establish a diagnosis (6). These techniques have been performed successfully and evolved into routine methods in Caucasian populations after ~20 years of clinical use (7–10). However, there are inadequate studies in Asian countries about MRI-guided biopsy (1, 11, 12), though MRI-guided biopsy is more valuable in China compared with ultrasound-guided biopsy or mammogram-guided biopsy because there are more dense areas in glandular tissue of Asian women. Therefore, we report our initial clinical experience with MRI-guided wire localization with subsequent surgical biopsy and MRI-guided biopsy of breast minimal lesions only visible on MRI in Chinese women.

## Methods

### Patient Population

This retrospective study was approved by the institutional review board of National Cancer Center/National Clinical Research Center for Cancer/Cancer Hospital, Chinese Academy of Medical Sciences and Peking Union Medical College, Beijing, China. We evaluated all the patients who underwent MRI-guided wire localization with subsequent surgical biopsy or MRI-guided breast biopsy from August 2013 to December 2017. Following the explanation of risks, benefits and proposed procedure, informed consent was obtained. All the examinations were repeated every 3–6 months within 1 year, every 6–12 months thereafter.

### MRI Acquisition Parameters

All the patients were scanned with a GE 1.5-Tesla MRI system in the prone position using a bilateral 8-channel phased-array breast coil. The target lesion site was disinfected before scanning, and the breast was fixed with a special puncture frame. MRI scans were acquired using the following sequences with an Achieve scanner: vibrant sequence; axial view; field of view, 36–38 cm; matrix size, 320 × 288; flip angle, 15; repetition time/echo time, 3.7/1.8 ms; slice thickness, 2.6 mm; and bandwidth 83.3 kHz. Unenhanced scans were performed before injection of a contrast agent. Continuous scans were performed without interruption 15 s after injection of the contrast agent. Using a high-pressure syringe, gadolinium-diethylenetriamine pentaacetic acid (Magnevist; Schering, Berlin, Germany) at a dose of 0.2 mmol/kg of body weight was injected at a velocity of 2.0 ml/s through the cubital vein. Approximately 20 ml of 0.9% normal saline was then injected with the same velocity to flush the vein.

### MRI-Guided Wire Localization and Biopsy

All interventions were performed by the same staff radiologist with more than 10 years of experience with breast imaging. All procedures were conducted with the same MRI equipment.

After the skin overlying the area of interest was prepared and draped in the usual sterile fashion, the patient was placed in a prone position in the 1.5-T MRI scanner supported with a dedicated biopsy compression device. Dynamic contrast-enhanced MRI (DCE-MRI) of the breast was obtained after contrast administration. The lesion depth was calculated based on the coordinates of the fiducial marker and the lesion. The patient was withdrawn from the magnet with the breast remaining in compression. After local anesthesia with 1% lidocaine, a scalpel incision was made in the skin. After insertion of the stylet introducer assembly to the appropriate depth, the stylet was removed from the breast, leaving the introducer within the breast and was replaced with an obturator to assist in MRI confirmation of the lesion location. After confirmation of the needle position and removal of the obturator, a 10-G vacuum-assisted biopsy (VAB) device was inserted into the introducer. Biopsy specimens were obtained, and the biopsy device was removed, followed by reinsertion of the obturator. A post-biopsy MRI was performed to determine if the lesion had been sampled. A compression bandage was wrapped around the patient's chest after the biopsy. To stop bleeding and to minimize the risk of hematoma formation, firm manual compression of the breast was performed for 10 min. The same procedure was used for MRI-guided wire localization.

The pathological diagnosis of 87 patients was confirmed by three or four pathologists with rich experience, including the pathological types, immunohistochemical and lymph node status of all suspicious lesions.

### Data Collection and Statistical Analysis

Eighty-eight suspicious lesions from 87 patients were retrospectively analyzed. This study included Breast Imaging-Reporting and Data System (BI-RADS) 4 or 5 lesions that were visible only on MRI. Clinical information including age, menstrual status, lesion location, histopathological results, and MRI characteristics of the targeted lesion (BI-RADS category, lesion size, and mass or non-mass enhancement) was recorded.

The chi-square test was used to compare lesion characteristics and histopathological results. SPSS version 15.0 (IBM, Chicago, USA) was used. The two-tailed *p* < 0.05 was set as the limit of statistical significance.

## Results

**Figure 1:**
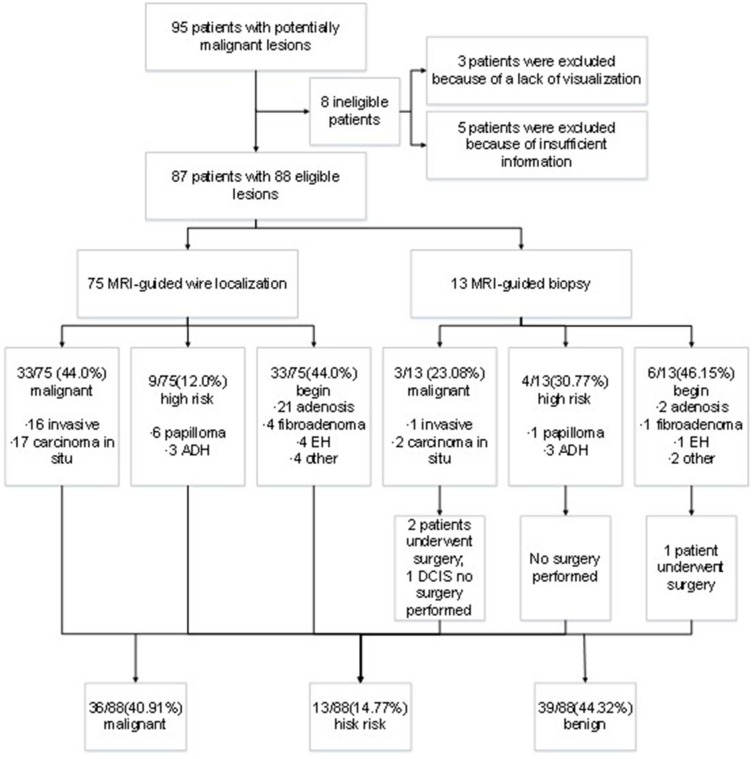
Flowchart of lesion outcomes.

A total of 95 patients underwent MRI-guided wire localization with subsequent surgical biopsy or MRI-guided breast biopsy from August 2013 to December 2017 at our institute. However, three cases had unsuccessful localization or biopsy attempts because of a lack of visualization of the initial biopsy target on the scheduled day of biopsy, and five cases were excluded from analysis for the lack of insufficient postoperative information. Ultimately, the remaining 87 patients were included into analysis. The outcomes of lesion was presented in Figure 1. The median age of patients was 50 years old ranging from 29 to 77 years old. There was no bleeding, hematoma, infection, or other adverse reactions after the MRI-guided biopsy. Meanwhile, no recurrent malignant breast tumor was found in all the patients during follow-up.

After biopsy or surgery, 36 of 88 lesions (40.91%) were found to be malignant, and 52 of 88 (59.09%) were benign. Seventeen of 88 lesions (19.32%) were invasive mammary carcinoma, and 19 (21.59%) were carcinoma *in situ* (Table 1). Of the 52 benign lesions, 23 of 88 (26.14%) were breast adenosis, five (5.68%) were epithelial hyperplasia (EH), five (5.68%) were breast fibroadenomas, seven (7.95%) were papillomas, six (6.82%) were atypical ductal hyperplasia (ADH), and six (6.82%) were other benign lesions (Table 1). All the lesions had morphologic features suspicious for or highly suggestive of malignancy according to the BI-RADS category of MRI (C4a = 44, C4b = 33, C4c = 9, and C5 = 2). Clinical characteristics of breast cancer patients with the MRI-guided wire localization and MRI-guided biopsy are summarized in Table 2.

**Table 1:** Pathology of lesions evaluated with MRI-guided biopsy or MRI-guided wire localization with subsequent surgical biopsy.

**Pathology**	**MRI-guided biopsy (*****n*** = 13)^*^		**MRI-guided wire localization with subsequent surgical biopsy (*****n*** = 75)^*^		**Total (*****n*** **= 88)**	
	***n***	**%**	***n***	**%**	***n***	**%**
Malignant	3	23.08	33	44.00	36	40.91
Invasive carcinoma	1	7.69	16	21.33	17	19.32
Carcinoma *in situ*	2	15.38	17	22.67	19	21.59
High risk	4	30.77	9	12.00	13	14.77
Papilloma	1	7.69	6	8.00	7	7.95
ADH	3	23.08	3	4.00	6	6.82
Benign	6	46.15	33	44.00	39	44.32
Adenosis	2	15.38	21	28.00	23	26.14
Fibroadenoma	1	7.69	4	5.33	5	5.68
EH	1	7.69	4	5.33	5	5.68
Other	2	15.38	4	5.33	6	6.82
Total	13	100	75	100	88	100

* *Number of tumors*.

*ADH, atypical ductal hyperplasia; EH, epithelial hyperplasia*.

**Table 2:** Clinical characteristics of patients who underwent MRI-guided biopsy or MRI-guided wire localization with subsequent surgical biopsy.

**Clinical characteristic**	***n***	**Benign (%)**	**Malignant (%)**	**χ^2^**	***p***
**Age (years)**					
≤40	15	12/15 (80.00)	3/15 (20.00)	5.00	0.08
40–60	55	33/55 (60.00)	22/55 (40.00)		
≥60	17	7/17 (41.18)	10/17 (58.82)		
**Menstrual status**					
Premenopausal	49	32/49 (65.31)	17/49 (34.69)	1.43	0.23
Postmenopausal	38	20/38 (52.63)	18/38 (47.37)		
**Imaging characteristic**					
Mass	39	26/39 (66.67)	13/39 (33.33)	1.21	0.27
Nonmass-like enhancement	49	27/49 (55.10)	22/49 (44.90)		
**Tumor size (cm)**					
≤1	29	21/29 (72.41)	8/29 (27.59)	7.60	0.55
1-2	29	17/29 (58.62)	12/29 (41.38)		
≥2	16	10/16 (62.50)	6/16 (37.50)		
NA	14	4/14 (28.57)	10/14 (71.43)		
**Bi-RADS**					
4a	44	28/44 (63.64)	16/44 (36.36)	4.03	0.26
4b	33	19/33 (57.58)	14/33 (42.42)		
4c	9	5/9 (55.56)	4/9 (44.44)		
5	2	0	2/2 (100)		

*NA, not available*.

**Figure 2:**
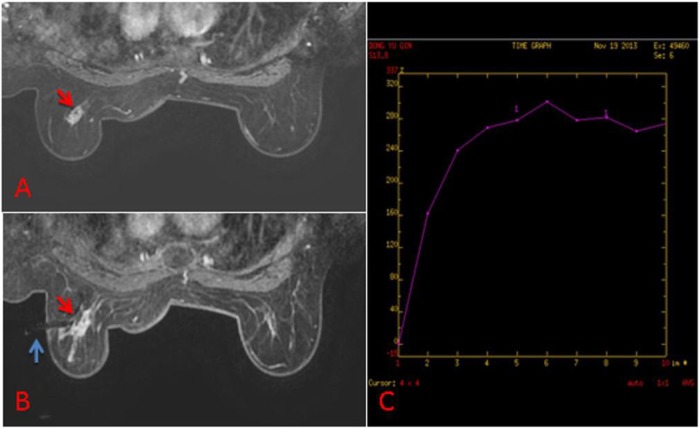
**(A)** 63-year old woman, T1-weighted fat suppressed contrast enhanced MRI showed a irregular mass at the left breast, which was suspicion for malignancy, and the BI-RADS category was considered as level 5. It was not found by ultrasound or mammography. **(B)** The lesion (red arrow) was performed by MR-guided biopsy (blue arrow), and it was confirmed to be invasive ductal carcinoma. **(C)** The dynamic contrast-enhanced time-signal intensity curve (TIC) was appeared as “fast-plateau type”.

**Figure 3:**
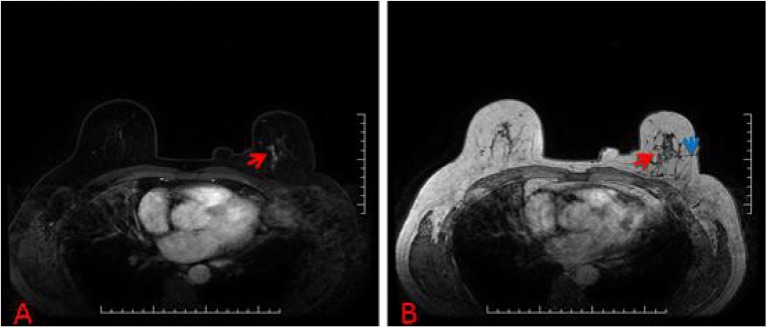
**(A)** 57-year-old woman, T1-weighted fat suppressed contrast enhanced MRI showed a suspicious lesion at the left breast, and the BI-RADS category was considered as level 4C. **(B)** The lesion (red arrow) that was revealed to be invasive ductal carcinoma after MRI-guided wire localization (blue arrow) with subsequent surgical biopsy (we chose T1-weighted MRI in order to show the lesion and wire location more clearly).

According the type of imaging, of the 88 lesions that were evaluated with MRI-guided biopsy (Figure 2) or localization (Figure 3), 39 of 88 (44.32%) were masses, including 13 malignant lesions and 26 benign lesions, and 49 of 88 (55.68%) had non-mass enhancement, including 22 malignant lesions and 27 benign lesions. However, there was no significant association between the malignancy rate and the type of lesion on MRI (*p* = 0.27). The mean size of all the lesions was 1.49 ± 0.91 cm; the mean lesion size was 1.57 ± 0.89 cm in malignant lesions vs. 1.46 ± 0.91 cm in benign lesions (*p* = 0.55). The probability of malignancy on MRI-guided biopsy or MRI-guided wire localization also increased with the BI-RADS category of the lesion of interest. In this study, the rate of malignancy for BI-RADS 5 lesions was 100% (2 of 2), compared with 44.44% for BI-RADS 4C lesions (4 of 9), 42.42% for BI-RADS 4B lesions (14 of 33), and 36.36% for BI-RADS 4A lesions (16 of 44).

## Discussion

The first MRI-guided clinical trials were reported in 1986 (13). Since then, MRI-guided clinical applications have been successfully applied to lesions in various anatomical regions, such as pancreatic, breast, prostate, lung tumor, and renal tumor lesions (14–18). After ~20 years of clinical use, MRI-guided breast biopsy has evolved into a routine method in Western European countries (19, 20). A small number of breast lesions cannot be detected by breast ultrasonography and mammography and can be identified only by MRI. Thus, the annual MRI scan is important for the breast cancer screening, especially for the women at high risk. However, according to the costs of more time and economy, MRI-guided procedures are reserved for lesions that are visible only on MRI and are judged to be suspicious and potential influence therapeutic decisions (21).

The malignancy rate in this study of women with MRI-visible breast lesions who underwent MRI-guided wire localization with subsequent surgical biopsy and MRI-guided biopsy of breast minimal lesions was 40.91% (in 13 patients who underwent MRI-guided biopsy, the malignancy rate was 23.08%, and in 74 patients with 75 eligible lesions who underwent MRI-guided wire localization with surgical biopsy, the malignancy rate was 44.0%), which is higher than the reported range of 20–35% (22–25). The main difference in this study was that the majority of patients (*n* = 74) underwent MRI-guided wire localization with subsequent surgical biopsy (the number of suspicious lesions was 75), and only 13 patients underwent MRI-guided biopsy. There may be several reasons for this phenomenon in this Chinese population. The most important reason is that there is currently no clinical experience MRI-guided breast biopsy in China, thus, Chinese patients may be more anxious compared with patients in Western countries, and when an occult lesion was found on MRI, most Chinese patients chose surgical treatment. Other reasons like the possible false negative rate of needle biopsy, the high self-paying price of MRI-guided biopsy also push breast surgeons and patients to chose MRI-guided wire localization with subsequent surgical biopsy. Thus, the majority of patients underwent MRI-guided wire localization with subsequent surgical biopsy in this study.

**Figure 4:**
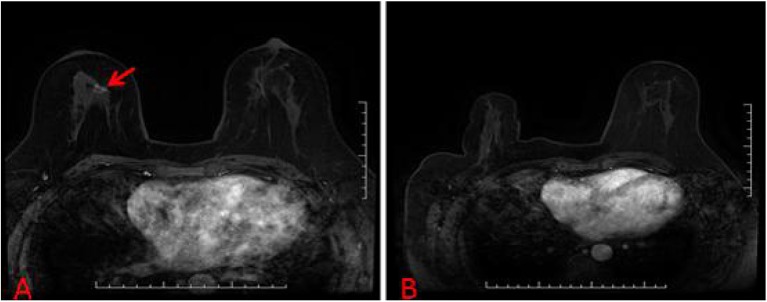
**(A)** A suspicious breast lesions (red arrow) that are visible only on MRI. **(B)** The patient with unsuccessful localization attempts because of a lack of visualization of the initial biopsy target on the scheduled day of biopsy.

MRI-guided biopsies have some chance of being canceled, and the reported rates of biopsy cancellation range from 8 to 13%; most often biopsies are canceled because of a lack of visualization of the initial biopsy target on the scheduled day of biopsy (26–28). In this study, three lesions (3.33%, 3/90) had unsuccessful localization or biopsy attempts because of a lack of visualization on the day of examination (Figure 4), but this rate is lower than the previously reported rate. These canceled biopsies might be a source of confusion and frustration for both clinicians and patients because of the small possibility of a missed malignancy, so a follow-up MRI at 6 months is recommended. Some studies have reported specifically on subsequent malignancies in patients with canceled MRI-guided biopsies (27–29). In the three patients who had canceled MRI-guided biopsies, no malignant breast tumors were found during follow-up until now.

There is currently no unified conclusion about the association between lesion morphology and malignancy rate. In our study, 55.68% (49 of 88) of the lesions had non-mass enhancement, and the cancer detection rate was 44.90% (22 of 49). The other lesions were masses, and the malignancy rate was 33.33% (13 of 39), which was lower than that for non-mass enhancing lesions. Rauch et al. (30) reported a greater malignancy rate for areas of non-mass enhancement (34%, 22 of 76) compared with masses (22%, 22 of 17). However, Han et al. (10) reported no significant difference in the malignancy rate of MRI-biopsied masses (34%) compared with that in non-mass enhancing lesions (27%).

Our research had some limitations. Firstly, though this is the largest sample study assessing the MRI-guided wire localization with subsequent surgical biopsy or MRI-guided breast biopsy in China, it is still limited by its relative small sample size and retrospective nature. Secondly, we did not correlate clinical stage and hormone receptors with imaging features. Finally, we did not consider the effect of breast size on the success rate of MRI-guided examination. Due to technical reasons, we rejected some patients with small breast volume in the early stage. With the accumulation of experience, fewer and fewer patients were excluded for this reason. In conclusion, large sample prospective studies which involve more information are warranted in the future.

Compared with ultrasound-guided biopsy or mammogram-guided biopsy, MRI-guided biopsy is more valuable in China because there are more dense areas in glandular tissue of Asian women. In addition, the rate of breast-conserving therapy in china is lower than that in Europe and American, the use of MRI-guided biopsy of breast minimal lesions could increase the rate of breast-conserving therapy in china. However, there are only few medical centers could performed this procedure due to lack of Asian specific breast coil which ought be smaller than Western one as well as insurance does not cover such procedure. Therefore, this study has an initiating significance in China.

## Conclusion

In conclusion, MRI-guided wire localization and MRI-guided biopsy can be safely, easily and effectively performed in occult breast lesions without major complications. After MRI-guided biopsy, follow-up within 6 months may be recommended because of the opportunity of underestimation of the disease. For occult lesions, MRI-guided biopsy is the only method that can confirm the diagnosis of suspicious enhancing breast lesions; however, this technique does pose some challenges.

## Data Availability Statement

The raw data supporting the conclusions of this article will be made available by the authors, without undue reservation, to any qualified researcher.

## Ethics Statement

Ethical review and approval was not required for the retrospective study on human participants in accordance with the local legislation and institutional requirements. The patients/participants provided their written informed consent to participate in this study.

## Author Contributions

JW wrote the manuscript and performed the statistical analysis. YS and JL contributed conception and design of the study. XM, ZX, and MZ organized the database. FY, XinW, and XiaW wrote sections of the manuscript. All authors contributed to manuscript revision, read, and approved the submitted version.

### Conflict of Interest

The authors declare that the research was conducted in the absence of any commercial or financial relationships that could be construed as a potential conflict of interest.
